# Synthesis, Characterization, and Biological Evaluation of Benzimidazole Derivatives as Potential Anxiolytics

**DOI:** 10.4103/0975-1483.66809

**Published:** 2010

**Authors:** DT Nannapaneni, Atyam VSSS Gupta, MI Reddy, Raidu Ch Sarva

**Affiliations:** *Department of Pharmaceutical Chemistry, Mallareddy College of Pharmacy, Dhullapally Village, Hyderabad - 500 014, India*; 1 *Department of Pharmacology, Joginpally B.R Pharmacy College, Yenkapally (P.O), Moinabad (Mandal), Ranga Reddy (Dist), Andhra Pradesh, India*

**Keywords:** Ammonium salts, anti-anxiety activity, benzimidazoles, carbonyl compounds, elevated plus maze model

## Abstract

The synthesized benzimidazoles compounds were prepared from the condensation reaction between *o*-Phenylenediamine and various carbonyl compounds, in the presence of ammonium chloride as a catalyst. Ammonium chloride is a commercial and environmentally benign catalyst. The yield of all benzimidazole derivatives was found to be in the range of 75 – 94%. The purity of the compounds was ascertained by melting point and TLC. The synthesized compounds were characterized by using IR,^1^H NMR, and MASS spectral data together with elemental analysis. The synthesized benzimidazole compounds were screened for acute and chronic anti-anxiety activity in Wistar rats by using an elevated plus maze model with standard Diazepam. The synthesized compounds Z_B_, Z_E_, Z_F_, Z_G_, and Z_H_ showed potent anti-anxiety activity when compared to the standard Diazepam. The compound Z_H_ exhibited a higher anti-anxiety activity when compared to other prepared benzimidazoles. The results were subjected to statistical analysis by using one-way ANOVA followed by the Tukey-Kramer test, to calculate the significance.

## INTRODUCTION

Benzimidazoles and its derivatives represent one of the most biologically active class of compounds, possessing a wide spectrum of activities and these are well-documented in literature. They show selective neuropeptides YY_1_ receptor antagonists,[1] potent inhibitors of TIE-2 and VEGFER-2 tyrosine kinase receptors,[2] antitumor agents,[3] gamma-amino butyric acid (GABA) agonists, and 5-HT_3_ antagonists.[4] Substituted benzimidazole derivatives have found commercial application in veterinarian medicine as anthelmintic agents and in diverse human therapeutic areas such as treatment of ulcers and antihistaminic.[5] Similarly, the general synthesis of benzimidazoles is by the condensation reaction of 1,2-phenylenediamine with carboxaldehydes, carboxylic acids,[6,7] or their derivatives[8,9] such as, chlorides, nitriles, and orthoesters, under strong acidic conditions, with high temperatures. Benzimidazoles have also been prepared on a solid phase to prove a combinatorial approach.[10] The most popular strategies for their synthesis utilize N-alkylation of an unsubstituted benzimidazoles.[11] Ammonium salts are inexpensive, commercially available reagents for few organic transformation reactions such as halogenation of aromatic compounds and synthesis of 3, 4-dihydropyrimidine-2(1H)-ones.[12] However, there are no reports of the use of ammonium salts as catalysts for the synthesis of benzimidazoles. In continuation, on the synthesis of heterocycles[13,14] and on the development of synthetic methodologies,[15–17] we herein report a facile method for the synthesis of benzimidazoles by the condensation of 1, 2-phenylenediamine with carbonyl compounds, in the presence of ammonium salts in very good yields. Anxiety is a psychological and physiological state characterized by cognitive, somatic, emotional, and behavioral components. These components combine to create an unpleasant feeling that is typically associated with uneasiness, fear or worry. Anxiety is a generalized mood or condition that occurs without an identifiable triggering stimulus. As such, it is distinguished from fear, which occurs in the presence of an observed threat. Additionally, fear is related to the specific behaviors of escape and avoidance, whereas, anxiety is the result of threats that are perceived to be uncontrollable or unavoidable. Some reports state that benzimidazoles possess anti-anxiety activity.[18] This observation prompted us to evaluate the synthesized benzimidazole derivatives for anti-anxiety activity

## MATERIALS AND METHODS

**Figure 1:**
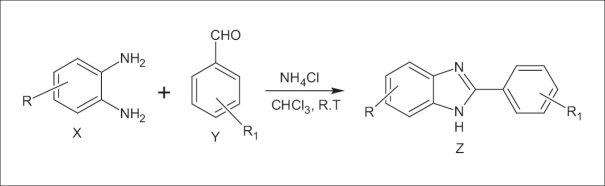
Ammonium halide catalyzed synthesis of 2-arylbenzimidazoles

All the chemicals and reagents used were of analytical grade and were procured from NICE Chemicals. It is known that the reaction of *o*-Phenylenediamine (OPDA) with carbonyl compounds, under strong acidic conditions, gives benzimidazoles, whereas, OPDA in the presence of β-ketoesters under neutral reflux conditions, gives benzodiazepin-2-ones, with the elimination of water and alcohol. Under acidic conditions, initially it forms ethyl β-2-amino aniline crotonate at room temperature and upon heating it gives 2-methyl-1H-benzo[d]imidazole instead of benzodiazepin-2-ones, with the elimination of ethyl acetate. Therefore, we have made an attempt to react OPDA with carbonyl compounds and b-ketoesters, with different ammonium salts to see the feasibility of the formation of compounds. To select favorable reaction conditions, we first examined the model reaction of 1, 2-phenylenediamine (1 mol) with benzaldehyde (1 mol) in the presence of NH_4_ Br (1 mol), under solvent-free conditions, at room temperature. The reaction was monitored by thin-layer chromatography (TLC, eluent Hexane/ethyl acetate 30/70) and 2-phenyl-1H-benzo[d]imidazole obtained in 20% yield. Similarly, the reaction was conducted in different solvents such as CH_3_CN, MeOH, CHCl_3_, ether, and DMF; CHCl_3_ was found to be the most suitable solvent that gave benzimidazole with 40% yield. Next, we carried out the same reaction with different ammonium salts such as NH_4_F, NH_4_Cl, NH_4_NO_3_, (NH_4_)_2_CO_3_, and (NH_4_)_2_SO_4_ in the presence of CHCl_3_, at room temperature; among these, NH_4_Cl (4 mol) gave 2-phenyl-1H-benzo[d]imidazole with 94% yield in 4 hours [Figure 1, Table 1]. The results, tabulated in Table 2, indicate the formation of benzimidazoles.

**Table 1:** Optimization of reaction conditions for the synthesis of 2-phenyl benzimidazole by the condensation of OPDA, with benzaldehyde, using various ammonium salts at room temperature in CHCl_3_

NH_4_X^a^	Time (hours)	Yield (%)^b^
NH_4_Br	4	86
NH_4_Cl	4	92
NH_4_F	5	72
(NH_4_)_2_SO_4_	12	78
(NH_4_)_2_CO_3_		82

a Reaction carried out with 4 mol of NH_4_X

b Isolated and unoptimized yields

**Table 2:**
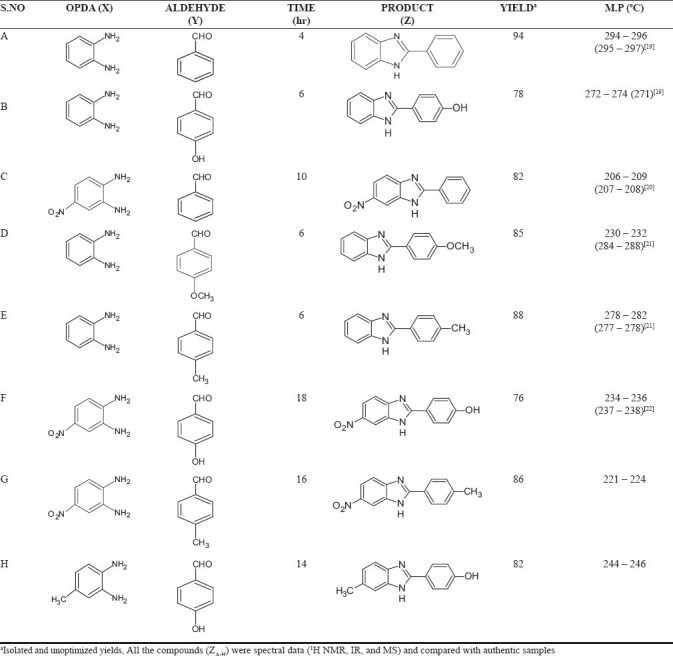
Synthesis of benzimidazole derivatives

The synthesized compounds were analyzed by NMR, Mass, and IR spectroscopy. ^1^H NMR spectra were recorded on a Varian Gemini 200- and 300-MHz instrument in CDCl_3_ and DMSO-d6 using Tetramethylsilane (TMS) as an internal standard. The mass spectra were measured on a Liquid Chromatography / Mass Spectrometry (LCMS) Agilent mass spectrometer. The IR spectra were recorded on a Nicolet 740 Fourier transform infrared (FTIR) spectrometer. The melting points were measured using a Buchi-510 apparatus and were uncorrected.

### Typical Experimental Procedure for the Synthesis of Benzimidazoles

Benzaldehyde (Y, 1 mmol) was added to a stirred solution of 1,2-phenylenediamine (X, 1 mmol) and NH_4_Cl (4 mmol) in CHCl3 (5 ml) for five minutes at room temperature. Stirring was continued for four hours. After completion of the reaction (TLC, eluent Hexane / ethyl acetate 30 / 70), the solvent was removed under reduced pressure and extracted with ethyl acetate (20 ml); the organic layer was washed with water (10 ml). Layers were separated and the organic layer was dried over sodium sulfate. The solvent was removed under reduced pressure and the crude product was subjected to column chromatography using petroleum ether = EtOAc (9:1), which gave 2-phenyl-1H-benzo[d]imidazole (Z_A_) as a solid in 94% yield.

### Spectral Data

#### 2-phenyl-1H-benzimidazole:

Solid; Molecular formula: C_13_H_10_N_2_, Yield-94%, m.p-296 °c;^1^H NMR: δ 6.06 (bs, 1H, NH), 6.82 (d, 2H, aromatic), 6.98 (d,2H, aromatic), 7.06 (t,1H, aromatic),7.28 (m, 2H, aromatic), 7.52 (m, 2H, aromatic), IR (KBr): 3426(-NH), 3042(Ar-CH), 1742, 1631(-C = N) cm^-1^; Mass (LCMS): *m/z* 195 (M ^+^ + H).

#### 4-(1H-benzimidazol-2-yl) phenol:

Solid; Molecular formula:C_13_H_10_N_2_O, Yield - 78%, m.p-271 °c; ^1^H NMR: δ 6.06 (bs, 1H, NH), 6.82 (d, 2H, aromatic), 6.98 (d,2H, aromatic), 7.21 (d,2H, aromatic),7.52 (d, 2H, aromatic), IR (KBr): 3379(-NH), 3211(-OH), 3078(-Ar-CH), 1461(C = N) cm-1; Mass (LCMS): *m/z* 211 (M ^+^ + H).

#### 6-nitro-2-phenyl-1H-benzimidazole:

Solid; Molecular formula:C_13_H_9_N_3_O_2_, Yield -82%, m.p-208 °c; ^1^H NMR: δ 6.08 (bs, 1H, NH), 6.90 (d, 2H, aromatic), 6.96 (d,2H, aromatic), 7.05 (t,1H, aromatic), 7.54 (d,1H, aromatic),8.12 (d, 1H, aromatic), 8.44 (s, 1H, aromatic), IR (KBr): 3211(-NH), 2984(Ar-CH), 1552(-NO_2_), 1529 (-C=N) cm^-1^; Mass (LCMS): *m/z* 240 (M ^+^ +H).

#### 2-(4-methoxyphenyl)-1H-benzimidazole:

Solid; Molecular formula:C_14_H_12_N_2_O, Yield -85%, m.p-285 °c; ^1^H NMR: δ 3.70 (d,3H, OCH_3_), 6.12 (bs, 1H, NH), 6.94 (d, 2H, aromatic), 6.98(d,2H, aromatic), 7.20 (d,2H, aromatic), 7.58 (d,2H, aromatic), IR (KBr): 3294(-NH), 3103(Ar-CH), 1184 (-OCH_3_), 1588(-C=N) cm-1; Mass (LCMS): *m/z* 225 (M ^+^ +H).

#### 2-(4-methylphenyl)-1H-benzimidazole:

Solid; Molecular formula:C_13_H_12_N_2_, Yield -88%, m.p-278 °c; ^1^H NMR: δ 2.54 (d, 3H, CH_3_),6.06 (bs, 1H, NH), 6.84 (d, 2H, aromatic), 6.96 (d,2H, aromatic), 7.18 (d,2H, aromatic),7.58 (d, 2H, aromatic), IR (KBr): 3346(-NH), 3024(Ar-CH), 2923(-CH_3_), 1575(-C=N) cm-1; Mass (LCMS): *m/z* 209 (M ^+^ +H).

#### 4-(6-nitro-1H-benzimidazol-2-yl) phenol:

Solid; Molecular formula:C_13_H_9_N_3_O_3_, Yield -76%, m.p-237 °c; ^1^H NMR: δ 6.08 (bs, 1H, NH), 6.74 (d, 2H, aromatic), 6.84 (d, 2H, aromatic), 7.45 (d,1H, aromatic), 8.02 (d,1H, aromatic),8.32 (s, 1H, aromatic), IR (KBr): 3737(-OH),3432(-NH), 3103(Ar-CH), 1562(C=N), 1532(-NO_2_) cm-1; Mass (LCMS): *m/z* 256 (M ^+^ +H).

#### 2-(4-methylphenyl)-6-nitro-1H-benzimidazole:

Solid; Molecular formula:C_14_H_14_N_3_O_2_, Yield -86%, m.p-223 °c; ^1^H NMR: δ 2.56 (d, 3H, CH_3_),6.10 (bs, 1H, NH), 6.80 (d, 2H, aromatic), 6.86 (d,2H, aromatic), 7.54 (d,1H, aromatic),8.08 (d, 2H, aromatic), 8.44 (s, 1H, aromatic), IR (KBr): 3402(-NH), 3054(Ar-CH), 1534(-C=N), 1524(-NO_2_) cm-1; Mass (LCMS): *m/z* 254 (M ^+^ +H).

#### 4-(6-methyl-1H-benzimidazol-2-yl) phenol:

Solid; Molecular formula:C_14_H_12_N_2_O, Yield -82%, m.p-245 °c; ^1^H NMR: δ 2.56 (d, 3H, CH_3_), 6.48 (bs, 1H, NH), 6.78 (d, 2H, aromatic), 6.92 (d,2H, aromatic), 7.48 (d,1H, aromatic), 8.10 (d,1H, aromatic), 8.44 (s,1H, aromatic), IR (KBr): 3455(-OH), 3274(-NH), 3212(Ar-CH), 2898(-CH_3_), 1534(-C=N) cm-1; Mass (LCMS): *m/z* 225 (M ^+^ +H).

### Pharmacology

Wistar rats weighing 200 ± 25 g, of either sex, were procured at least two weeks prior to the study. The animals were housed in polycarbated cages under conditions of constant temperature (22 ± 2 °c) and humidity, under a 12-hour light / dark schedule, The animals were allowed free access to a standard diet and water, so they could acclimatize to the new environment.

### Acute oral toxicity studies

The aim of this study is to determine the lethal dose. In this study the testing drug was administered in a single dose, using the oral route. The dose was increased in a graded manner. LD_50_ in the acute toxicity test was observed at the dose of 400 mg/kg-bw. Therefore, one-tenth of the preceding dose (200 mg/kg-bw) was selected for the study, that is, 20 mg/kg-bw. This 20 mg/kg-bw testing dose did not have any impact on the normal locomotion of the animal, which was tested by administering the same dose to the animals, using the oral route. The locomator activity was assessed in an actophotometer.

### Anti-anxiety activity

In this activity, the elevated plus maze model was used. For this model, Wistar rats were divided into 10 groups of six animals each. Group-I (control) animals were administered the vehicle, Group-II, Group-III, Group-IV, Group-V, Group-VI, Group-VII, Group-VIII, and Group-IX animals were administered the benzimidazole derivatives of Z_A_ – Z_H,_ respectively, with a dose of 20 mg/kg-bw (p.o), and Group-X was administered diazepam 2 mg/kg-bw (p.o), for 1 day in case of acute study and for 10 days in case of chronic study. The number of entries and time spent in the open and closed arms of the elevated plus maze, using rats, was observed in acute and chronic studies for 1day and 10 days, respectively. Diazepam was used as a reference standard. The experiment was conducted in a sound attenuated room. In an acute study, the animals of all groups were treated with the respective drugs 30 minutes prior to the experiment. In a chronic study, animals of all groups were treated with the respective drugs for 10 days, and on the tenth day, treatment was given 30 minutes prior to the experiment. In both acute and chronic studies, each rat was placed in the center of the maze facing one of the enclosed arms, and during a ten-minute session the following parameters were noted; Number of entries into the open arm, number of entries into the closed arm, time spent in the open arm, time spent in the closed arm, and total number of entries into the open and closed arms.[23,24]

### Statistical Analysis

The values were expressed as mean ± SEM for six animals. The results were subjected to statistical analysis by using one-way ANOVA followed by Tukey-Kramer test, to calculate the significant difference, if any, among the groups. *P* < 0.05 was considered as significant.

## RESULTS

The compounds (Z_A_ – Z_H_) were obtained by the reaction between aldehyde and o-phenylediamine in the presence of ammonium salts according to Figure 1. The synthesized compounds were confirmed by thin layer chromatography (TLC), Melting Point (mp), IR, ^1^H NMR, and mass spectroscopy (MS) spectral analysis. The yields and melting points for all the synthesized compounds are listed in Table 2. The titled compounds were confirmed by IR spectral data showing characteristic bands at 1384 – 3200 cm^-1^, indicating the presence of –NO_2_ and –OH stretching; and sharp bands, ranging between 1680 – 1750 cm^-1^, indicating the presence of C = N. Compounds Z_A_ – Z_H_ were confirmed by stretching at 3500 cm^-1^, due to the presence of –NH. Compounds Z_A_ – Z_H_ were confirmed by ^1^ H NMR spectral analysis. The NMR proton peak at 6.00 – 6.18 ppm revealed the presence of –NH. Further appearance of the molecular ion peak at 225 (m + 1) and 209 (m + 1) confirmed the structure of Z_D_ and Z_E_. The synthesized compounds Z_B_, Z_E_, Z_F_, Z_G_, and Z_H_ were found to have potent anti-anxiety activity. Compound Z_H_ exhibited more activity when compared to other prepared benzimidazoles.

### Anti-anxiety activity

In the acute and chronic studies of elevated plus maze models [Tables 3 and 4], the number of entries into the open and closed arms and the time spent in the open arm were increased; and time spent in the closed arm was decreased in synthetic preparation-treated animals when compared with the control animals, which was comparable with that of the reference standard, diazepam.

**Table 3:** Effect of synthetic preparation on number of entries and time spent in elevated plus maze in acute study

Treatment group	Open arm entries	Closed arm entries	Total entries	Time spent in open arm	Time spent in closed arm
Control	2.5 ± 0.4282	2.16 ± 0.3073	4.66 ± 0.6667	16.83 ± 1.352	583.16 ± 1.352
Z_A_	3.83 ± 0.3070	3.66 ± 0.4216	7.5 ± 0.7188	23 ± 1.673	577 ± 1.673
Z_B_	4.50 ± 0.5627	4.33 ± 0.7149*	8.83 ± 1.249*	35.5 ± 1.310*	564.5 ± 1.310*
Z_C_	4.16 ± 0.4773	3.83 ± 0.4773	8 ± 0.8563	19.83 ± 1.249	580.16 ± 1.249
Z_D_	2.66 ± 0.3333	3 ± 0.2582	5.66 ± 0.4216	17.83 ± 1.579	582.16 ± 1.579
Z_E_	5.16 ± 0.4773*	4.83 ± 0.3073**	10 ± 0.6831**	92.16 ± 6.085***	507.83 ± 6.085***
Z_F_	5 ± 0.3651*	5 ± 0.4475***	10 ± 0.6831**	101.66 ± 3.432***	498.33 ± 3.432***
Z_G_	5.16 ± 0.6009*	5 ± 0.3651***	10.16 ± 0.9098**	104.33 ± 3.658***	495.66 ± 3.658***
Z_H_	5.66 ± 0.7601**	5.66 ± 0.3333***	11.33 ± 1.685***	115.33 ± 4.835***	484.66 ± 4.835***
Std (Dizepam)	6.5 ± 0.4282***	6.83 ± 0.4773***	13.33 ± 0.8028***	134.16 ± 4.430***	465.83 ± 4.430***

Significance: All values are given in mean ± SEM, n = 6. One way ANOVA followed by Tukey’s multiple comparison test

* *P* < 0.05 vs. control

** *P* < 0.01

*** *P* < 0.001

**Table 4:** Effect of synthetic preparation on number of entries and time spent in elevated plus maze model in chronic study

Treatment group	Open arm entries	Closed arm entries	Total entries	Time spent in open arm	Time spent in closed arm
Control	3.83 ± 0.3073	3.83 ± 0.3073	7.66 ± 0.4216	19 ± 1.211	581 ± 1.211
Z_A_	4.83 ± 0.3073	4.16 ± 0.3073	9 ± 0.4472	24.5 ± 1.784	575.5 ± 1.784
Z_B_	6.66 ± 0.7149**	7 ± 0.577**	13.66 ± 1.229***	85.5 ± 2.291***	514.5 ± 2.291***
Z_C_	5.16 ± 0.4773	5.16 ± 0.4773	10.33 ± 0.8433	25.33 ± 1.60	574.66 ± 1.60
Z_D_	3.83 ± 0.3073	4.16 ± 0.4773	8 ± 0.6831	25.33 ± 2.275	574.66 ± 2.275
Z_E_	6 ± 0.2582*	6 ± 0.3651	12 ± 0.4472*	97.83 ± 4.003***	502.16 ± 4.003***
Z_F_	6.16 ± 0.3073*	6.5 ± 0.4282*	12.66 ± 0.646**	106.16 ± 2.040***	493.83 ± 2.040***
Z_G_	6.33 ± 0.6146**	6.33 ± 0.8433*	12.66 ± 1.406**	111.66 ± 2.246***	488.33 ± 2.246***
Z_H_	6.83 ± 0.7032***	6.83 ± 0.6009**	13.66 ± 1.229***	121 ± 3.307***	479 ± 3.307***
Std (Dizepam)	7.5 ± 0.4282***	7.83 ± 0.6009***	15.33 ± 0.9545***	136.33 ± 2.985***	463.66 ± 2.985***

Significance: All values are given in mean ± SEM, n = 6. One way ANOVA followed by Tukey’s multiple comparison test

* *P* < 0.05 vs. control

** *P* < 0.01

*** *P* < 0.001

## DISCUSSION

Several researchers reported a synthesis of benzimidazole derivatives, but in our present study we synthesized the benzimidazole derivatives by using ammonium salts as catalysts, which were inexpensive and decreased the reaction time, with very good yields. This method could be easily practiced in laboratories within the stipulated time.

In the evaluation of anti-anxiety activity, the experimental model used in our study was an elevated plus maze. This was based on the assumption that unfamiliar, non-protective, and brightly lit environmental stress provoked inhibition of normal behavior. This normal behavioral inhibition was further augmented in the presence of fear or an anxiety-like state.[24] The elevated plus maze test was a well-established animal model for testing anxiolytic drugs. A known anxiolytic drug, diazepam, was used as the standard, which is one of the well-recognized anxiolytic drugs. In this model, the changes that occurred due to anxiety were, decreased time spent in the open arm, increased time spent in the closed arm, and decreased number of entries between the arms, than that which was observed in the control animals.

After treatment with the synthetic preparation, at a dose of 20 mg/kg-bw, the compounds Z_B_, Z_E_, Z_F_, Z_G_, Z_H_, and the standard, significantly increased the time spent in the open arm, decreased the time spent in the closed arm, and increased the number of entries between the arms, in the acute study [Table 3]. All these may be due to decreased fear, increased exploratory behavior, and the behavioral disinhibitory effect[24] of the standard (Diazepam) and synthetic preparation. Similar findings were observed in the chronic study also [Table 4]. This may be due to the same mechanism, as stated earlier in the acute study.

## CONCLUSION

The present study describes a simple, inexpensive, and easy method for synthesis of benzimidazole derivatives in a stipulated time, without using any drastic conditions. The yield of all benzimidazole derivatives were found to be in the range of 75 – 94%. The purity of the compounds were ascertained by a melting point and TLC. The assigned structure was further established by IR,^1^ HNMR, and MS spectral studies.

The acute and chronic studies for anti-anxiety activity of the synthesized compounds were screened using elevated plus maze method in Wistar rats. Diazepam was used as the reference drug. In the prepared benzimidazole derivatives, it seemed that the compounds Z_B_[4-(1*H*-benzimidazol-2-yl) phenol], Z_E_[2-(4-methylphenyl)-1*H*-benzimidazole], Z_F_[4-(6-nitro-1*H*-benzimidazol-2-yl) phenol], Z_G_[2-(4-methylphenyl)-6-nitro-1*H*-benzimidazole], and Z_H_[4-(6-methyl-1*H*-benzimidazol-2-yl) phenol] showed potent activity when compared to the standard drug diazepam. The compound Z_H_[4-(6-methyl-1*H*-benzimidazol-2-yl) phenol] exhibited the highest anti-anxiety activity when compared to the other prepared benzimidazole compounds.

From the present study, it can be concluded that the benzimidazole derivatives can potentially be developed into useful anti-anxiety agents, which can prompt future researchers to synthesize a series of benzimidazole derivatives containing a wide variety of substituent’s, with the aim of producing a novel heterocyclic system, with enhanced activity.
